# The cost of blind spots: How the 2025 USAID closure weakened global outbreak detection

**DOI:** 10.3389/fpubh.2026.1887795

**Published:** 2026-09-03

**Authors:** Alex Mirugwe

**Affiliations:** School of Public Health, Makerere University, Kampala, Uganda

**Keywords:** Africa CDC, disease surveillance, donor transitions, Ebola Bundibugyo, global health security, PHEIC, U.S. foreign aid, USAID

## Background on the USAID closure

In early 2025, shortly after assuming office, the Trump administration ordered a comprehensive review of U.S. foreign assistance. This led to an immediate pause on new obligations and disbursements, followed by stop-work orders and the termination of approximately 83% of USAID's programs. By mid-2025, the agency was effectively dismantled, with remaining functions absorbed into the Department of State amid substantial staff reductions and contract cancellations. The administration cited the need to eliminate waste, refocus on core national interests, and ensure greater accountability and efficiency in foreign aid spending (1, 2).

For decades, USAID had played a central role in building and sustaining global health security. It funded surveillance networks in zoonotic hotspots, supported laboratory strengthening, trained frontline health workers, and enabled rapid sample transport and testing. These investments, often implemented in partnership with the CDC, WHO, and local governments, had repeatedly demonstrated their value by shortening outbreak detection and response times in challenging environments (3). A single flagship activity, USAID's STRIDES program, represented an estimated $245 million investment in partner-country disease-detection capacity. Independent government evaluations had previously found that the impact of such surveillance investments on outbreak detection was rarely assessed in the peer-reviewed literature, a gap that also limits precise attribution of the 2026 outbreak delays discussed below (4).

## Surveillance capacity and global health security

### The 2026 Bundibugyo Ebola outbreak

In mid-May 2026, health authorities in the Democratic Republic of the Congo confirmed an outbreak of Ebola disease caused by the Bundibugyo virus in Ituri Province. As of the WHO's initial Disease Outbreak News notice on May 17, 2026, the outbreak had been linked to roughly 12 laboratory-confirmed cases and around 500 suspected cases. WHO's subsequent weekly situation reports revised these figures as retrospective testing cleared a backlog of samples: 85 confirmed cases and 746 cumulative suspected cases, including 176 deaths among suspected cases, by May 21, and 550 confirmed cases across Ituri, North Kivu, and South Kivu provinces by June 7 (5, 6). This progression, in which suspected

cases are successively laboratory-confirmed or excluded, accounts for the wide ranges reported in early coverage rather than reflecting inconsistent case counting. The virus also spread to Uganda, where 20 confirmed cases and 2 deaths linked to travelers from the DRC had been recorded by mid-July, with no new cases reported since June 21. On May 17, the WHO declared the event a Public Health Emergency of International Concern (7).

This strain, rarer than the more familiar *Zaire ebolavirus*, lacks approved vaccines or specific treatments, complicating containment. The outbreak likely circulated for several weeks before confirmation, gaining momentum amid conflict, population displacement, and strained local health systems. Initial testing protocols focused on more common strains may have contributed to the delay in recognition (8).

Public health experts have noted that the erosion of USAID-supported networks likely played a role in the slower early response. Prior to 2025, U.S.-backed programs in eastern DRC provided critical community surveillance, laboratory logistics, and rapid reporting capabilities that had helped contain previous outbreaks more swiftly. With many of these disrupted, local authorities faced greater challenges in detecting and verifying the unusual cluster of severe illnesses in a remote, insecure region. While zoonotic spillover from animal reservoirs and ongoing conflict remain the primary drivers of emergence, the loss of established early-warning infrastructure may have contributed to wider transmission before full international mobilization (9, 10).

This account remains circumstantial: the manuscript is not aware of published data quantifying which specific surveillance activities, laboratory contracts, or field staff positions in eastern DRC were interrupted by USAID's restructuring, nor of a formal before-after comparison of detection capacity in the affected health zones. For context, a peer-reviewed analysis of outbreaks reported to WHO's African Region between 2017 and 2019 found a median time to detection of 8 days (interquartile range 2–28), with detection times improving year over year (11). The multi-week interval before the Bundibugyo outbreak was confirmed sits well outside that historical range, though attributing this specific delay to the loss of any single donor's programs, rather than to the conflict-affected and remote setting in which the outbreak emerged, would require activity-level surveillance data that are not yet publicly available.

### Policy implications

The concurrent 2026 events reveal the practical consequences of reduced surveillance capacity. Critics, including former officials and global health analysts, had warned that abrupt cuts would create blind spots in precisely the regions where new threats often emerge first. Projections indicated not only setbacks in controlling endemic diseases but also slower detection of emerging ones. In an interconnected world, delays in one location can lead to importations and wider risks, as evidenced by travel-related cases in this outbreak (12).

Defenders of the policy emphasize that many programs suffered from inefficiencies and that remaining functions could be managed more effectively under the State Department. They argue that outbreaks stem primarily from local ecological and social factors, and that other actors, such as the Africa CDC and WHO, are actively responding. These points are valid. Foreign assistance has long required scrutiny for sustainability and results, and diversified funding can reduce dependency on any single donor. In this very outbreak, the Africa Centres for Disease Control and Prevention (Africa CDC) declared a Public Health Emergency of Continental Security and launched a joint response plan with WHO, illustrating the growing capacity of regional institutions to lead health-security responses (13, 14).

This regional stepping-up should be read against a backdrop of structural donor dependence that predates the 2025 closure. Sub-Saharan African governments devoted only around 7.2% of national budgets to health in recent years, compared with an average of 12.4% in other regions, while development assistance for health is projected to grow more slowly than the economies it supports (15). A systematic review of donor-funded health programs across Africa found that fewer than four in five continued to operate beyond their original funding period, underscoring that reliance on any single external donor, including USAID, was already a recognized vulnerability before this closure (4). Strengthening domestic financing and regional institutions such as Africa CDC is therefore not merely a response to USAID's withdrawal but a longer-standing priority that this episode makes more urgent (2).

Nevertheless, historical performance data show that U.S.-supported systems provided measurable speed and scale in early detection that partners have not fully replaced in the short term. Early containment remains far less costly than large-scale responses or potential pandemic-level events.

## Lessons for future pandemic preparedness

A pragmatic path forward involves restoring targeted surveillance and laboratory capacities in high-risk zones through hybrid models that combine bilateral efforts with multilateral partners. Greater emphasis on building resilient, locally owned systems, alongside rigorous accountability metrics, could address efficiency concerns while preserving essential early-warning functions. Treating global health security as an enduring national interest, rather than subject to abrupt shifts, would better protect both humanitarian goals and U.S. security.

Outbreaks will continue to arise from the interplay of nature, human behavior, and environmental pressures. The question is whether societies confront them with robust detection systems or with avoidable gaps in visibility. The events of May 2026 suggest that preserving smart, accountable surveillance infrastructure is not merely an expense but a necessary investment in collective safety.
